# Residual Error Based Anomaly Detection Using Auto-Encoder in SMD Machine Sound

**DOI:** 10.3390/s18051308

**Published:** 2018-04-24

**Authors:** Dong Yul Oh, Il Dong Yun

**Affiliations:** 1 Department of Digital Information Engineering, Hankuk University of Foreign Studies, Yongin 17035, Korea; dyoh@hufs.ac.kr; 2 Division of Computer & Electronic System Engineering, Hankuk University of Foreign Studies, Yongin 17035, Korea

**Keywords:** auto-encoder, machine sound, anomaly detection, SMD, unsupervised learning

## Abstract

Detecting an anomaly or an abnormal situation from given noise is highly useful in an environment where constantly verifying and monitoring a machine is required. As deep learning algorithms are further developed, current studies have focused on this problem. However, there are too many variables to define anomalies, and the human annotation for a large collection of abnormal data labeled at the class-level is very labor-intensive. In this paper, we propose to detect abnormal operation sounds or outliers in a very complex machine along with reducing the data-driven annotation cost. The architecture of the proposed model is based on an auto-encoder, and it uses the residual error, which stands for its reconstruction quality, to identify the anomaly. We assess our model using Surface-Mounted Device (SMD) machine sound, which is very complex, as experimental data, and state-of-the-art performance is successfully achieved for anomaly detection.

## 1. Introduction

Sound is one of the key aspects to identify an object. Many studies analyzing and classifying sound events have been proposed and are still underway with many competitive goals. Furthermore, recent developments in deep learning and machine learning are becoming more popular than conventional methods including rule-based techniques [1,2]. As GPUs evolve and big data become readily available, the development speed for deep learning has increased exponentially, and many neural network models for sound classification have been proposed [3,4,5] using Convolution Neural Networks (CNNs) [6]. Existing methods and deep learning algorithms solve the same problem such as classification, using given data ranging from raw data to extracted data (features of interest), and they differ only in which methods are used. For instance, Dai et al. [5] use the raw waveforms of a given signal; Hershey et al. [3] and Gorin et al. [4] use frequency variations over time, such as STFT; and also Vu et al. [7] use recurrent neural networks [8].

Industrial machines, such as semiconductor assembly equipment and automotive component assembly equipment, tend to stop the entire production line once they detect faults or break down, which often leads to large losses. Thus, to determine machine faults in advance, several approaches have become available in various domains, including computer vision [9,10,11]. In particular, in an environment where humans cannot constantly check inside machines, including motors, noise can be used as a useful alternative to detect abnormal situations. Many approaches to conduct fault detection through machine operating sounds and STFT have been previously proposed [12,13,14]. These have been considered using a very cost-effective modality that uses the machine sound without other complicated electrical signal sensors. Since auditory perception is quite sensitive to ambient noise, most sensors are inside the machine, closely attached to the target that they constantly observe. In medicine, on the other hand, ambient noise is not very common, and many models have been proposed to verify patient health by analyzing the heart [15] and snoring sounds [16]. The same modality can be divided into several domains, ranging from raw waveforms to spectrograms with frequency variations over time, and these are usually obtained via STFT. In addition, post-processing is also used with a spectrogram that takes human audible frequencies into account and reduces the dimension to avoid unnecessary frequencies [17,18]. This is referred to as Mel-Frequency Cepstral Coefficients (MFCCs) and has also been utilized to describe the feature of interest and to apply machine learning algorithms [19].

However, most solutions include an approach that learns both abnormal and normal sounds in classification. These generally classify at least two classes, including binary SVM, thereby the model only focuses on hyper-planes that better distinguish the classes. As such, general classification models can only discriminate among the finite set of predefined classes, which outputs the wrong prediction when the data are measured well outside of the expected range or are not similar to the training set at all. For this reason, existing models often suffer from the lack of strong prior information for unknown abnormalities. A label that includes all other data can possibly solve this problem. However, “all other data” is highly ambiguous. Even when the model is successfully trained, it is difficult to define how abnormal sounds are different from the normal ones by their classification scores, as well as to obtain meaningful measures from them. Thus, we recast this problem as a regression-based classification via manifold-learning within a deep learning framework. The proposed model has succeeded not only in achieving high performance on more complex and noisy data than simple and monotonous data, but also in overcoming the limitations of the existing methods mentioned above.

The key contributions of our work are summarized below:

- We developed cost-effective anomaly detection from the given noise within an auto-encoder framework, which classifies outliers without expensive annotation for anomalies.
- We introduced a straightforward method to determine anomalies based on high reconstruction errors, which indicates how close data are to outliers or whether data approach the outliers.
- We successfully detected the abnormal sound of complex assembly machines, such as aging and intermittent noise. These also include differences in the assembly parts and the one whose grease has been removed.

## 2. Proposed Approach

**Figure 1:**
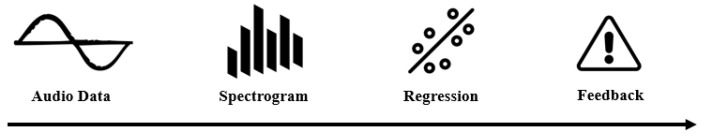
Overall schematic of the proposed model.

In this section, our approach is described in detail based on the overall schematic of the model, depicted in Figure 1. The basic steps for data pre-processing will be introduced in Section 2.1, and the neural network-based regression method for the anomaly detection will be discussed in Section 2.2 and Section 2.3. We do not discuss the feedback because it only involves the user’s response or request after detecting the anomaly using our approach.

### 2.1. Data Pre-Processing

The proposed approach detects an abnormal situation or monitors the state of the machine with its operation sound. There are two typical data types for audio signals, amplitudes in the time domain (raw waveform) and those in the frequency domain (spectrogram). Without any data pre-processing method, using raw waveforms directly is a more difficult problem for the neural network. The neural network must learn the frequency bands of the raw data by itself, because the data that we use are the audio signals. Theoretically, the difference between abnormal and normal data is observed in the frequency domain. Therefore, the network needs many kernels corresponding to each frequency band to analyze the different band. The deep learning approach somehow fits the model during the training process, but most of the time, it is desirable to provide some small hints instead of entirely relying on its system. The data pre-processing can be regarded as one of the guided trainings. It also pre-defines the features that the neural network should learn, which allows the model to converge more quickly and efficiently. Thus, we adopted the spectrogram through a Short-Time Fourier Transform (STFT). Note that the neural network model without STFT needs some more layers instead of STFT, and this is not quite efficient when we change the target machine. When we extend the model to other production lines, the model should be re-trained by the normal operation data of the target machine. Therefore, in terms of training time, adding layers is not efficient.

Mel-Frequency Cepstral Coefficients (MFCC) comprise a feature domain that is widely used in speaker recognition and speech recognition [20], and it has been successfully applied in machine diagnosis [17,18]. However, since the frequency changes from the Hz to mel scale by its mel-frequency wrapping filters and discrete cosine transform, the details are often lost even if it focuses on the significant component and discards background noises from the given signals. In addition, it is highly unlikely to be effective with very complex machine defects where humans cannot easily make distinctions due to the limitations in auditory ability. Therefore, we considered all frequency domain ranges via STFT in our input for the deep learning model with a powerful performance.

**Figure 2:**
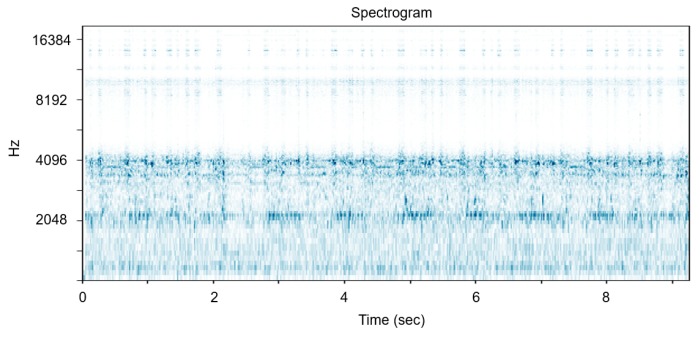
Spectrogram from short time Fourier transform of the Surface-Mounted Device (SMD) machine sound with sampling rate = 44,100 Hz, window = Hann, window size = 2048 samples and overlap length = 512 samples.

**Figure 3:**
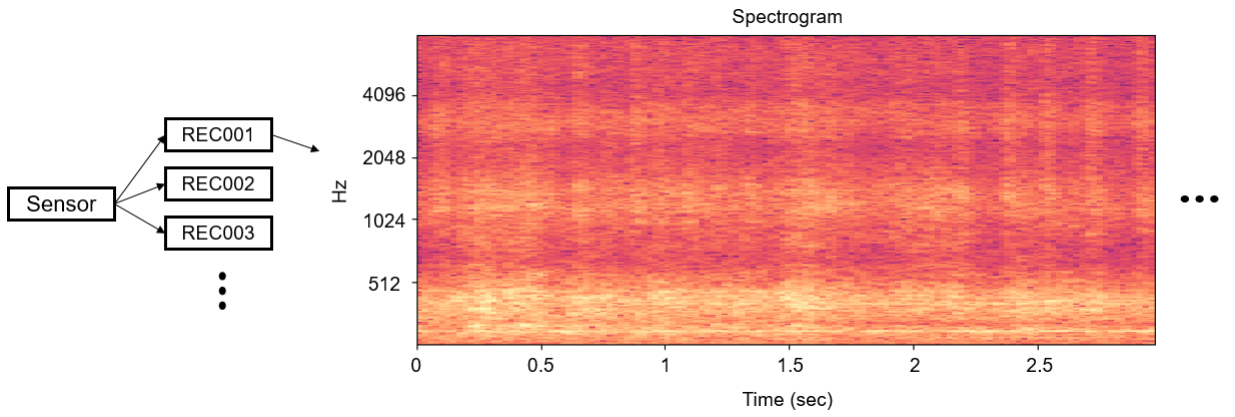
Example of STFT for recorded data from the SMD machine with sampling rate = 16,300 Hz, window = Hann, window size = 2048 samples and overlap length = 512 samples.

In terms of the hyper-parameters in STFT, the higher the sampling rate, the higher the maximum frequency that can be analyzed. As we get more details of the frequency bands, the performance often gets improved; however, the data processing burden increases at the same time. The motivation of this research is that the professional manager can successfully grasp the anomaly by the operation sound. We designed the system especially when the experts cannot monitor the machine constantly, and the goal of this research is to detect abnormalities and inform the managers. Therefore, we concluded that the optimal sampling rate is close to the audible frequency, 16 kHz. We have experimented on other values such as 44 K = kHz and detected that there is almost no available information in the high frequency bands, as shown in Figure 2. Most of the energy is concentrated on audible frequency. In addition, we have experimented with smaller window sizes and set the overlap length as a quarter and a half of each window size. However, all these cases showed that the frequency resolution was too small for the network to capture the frequency difference between abnormal and normal data and that smaller overlap could not capture the intermittent noise. As a result, all STFT hyper-parameters depicted in Figure 3 have been determined through several experiments.

After applying the STFT to the audio files that we want to classify, we segmented the STFT results about every 1.5 s. The segmentation interval also has been experimentally determined upon the fact that experts generally need sounds at least 2 s long in complex machines to identify their states. However, the segmentation interval can be adjusted according to the equipment type. In addition, the DC component was first removed, and we normalized all spectrograms because the amplitude differences depending on the recording volume could potentially exist, and this represents different ranges of the feature distribution. The height of the spectrogram matrix shown in Figure 3 indicates that the frequency resolution is 1024. Finally, we split the spectrogram into 32 columns for all data. More details about the acquisition process of audio data and the use of spectrograms in our model will be introduced in the next sections.

### 2.2. Auto-Encoder-Based Architecture

General classification models can only discriminate among the finite set of classes predefined. This approach needs at least two categories, “normal” and “abnormal”. However, as previously described in Section 1, there are too many variables to define anomalies, and the human annotation for a large collection of abnormal data labeled at the class level is very labor-intensive. It often suffers from the lack of prior information for unknown abnormal classes, and they are practically impossible to be predicted. When the abnormal data are not easily available, the neural network cannot perform very well because of the data imbalance. All these problems are due to the fact that we attempt to solve them by a classification problem and to select a better classifier. As a result, we propose to recast this problem as a regression-based abnormality decision via manifold learning with the auto-encoder.

**Figure 4:**
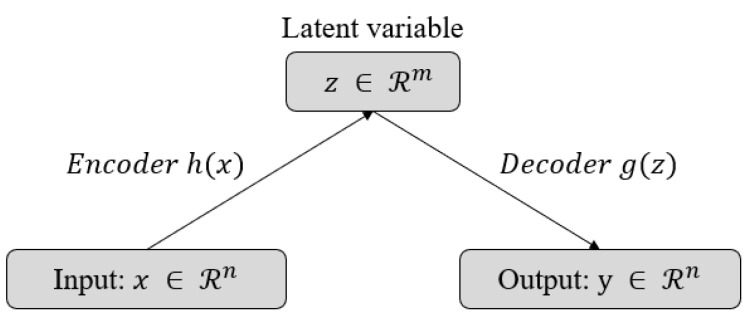
Basic framework for general auto-encoders. The latent variable *z* is the consequence of the dimensionality reduction of the auto-encoder. The data that the encoder and the decoder have been trained with must be able to be represented as a latent vector and be well reconstructed.

The technical details of the proposed model are all based on a general auto-encoder (see Figure 4). The Variational Auto-Encoder (VAE) [21], which is a well-known extension model of the auto-encoder, is a probabilistic model that approximates the likelihood and a posterior of the KL-divergence with variational inference. However, it is difficult to view the proposed model as a generative model similar to VAE. We do not require any generative models such as interpretation or interpolation on a low-dimensional latent space that has high-level abstract features. In other words, our measures for anomalies or outliers do not require prior knowledge for the latent variable *z*, unlike VAE. Since the VAE series is easy to handle and has constraints on prior knowledge, such as Gaussian or the exponential family, the performance of the reconstruction for the data with a high variance is lower than that of a general auto-encoder. Therefore, we adopted a general auto-encoder that regularizes the data well enough in our problem.

**Figure 5:**
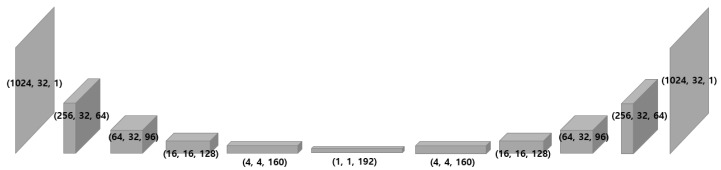
Overall structure of our model and geometrical change of an input as passing layers. The input shape is set as height = 1024, width = 32, channel = 1, as we split the spectrograms into 32 columns where the frequency resolutions is 1024.

The auto-encoder is a network with the same input and output. The objective function to train the network should aim to reduce the difference between the reconstruction and its original spectrogram. The mean squared error is suitable for this case since it is not a classification problem, but rather a regression problem. As depicted in Figure 5, the model produces an output, which is the same as the input, by decoding (reconstructing) the encoded (dimensionality reduced) information. Without an internal transformation process such as dimensionality reduction and expansion, the model is very likely to converge to an identity matrix that can easily achieve the objective function. Therefore, this process can be considered the curve fitting in the low-dimensional space. Note that all spectrograms are normalized to be zero-centered, which transforms the input into the regions that can utilize the non-linearity of the proposed model and not get saturated. Further implementation details of the proposed model can be found in Appendix A.

The most compressed component in the middle of the network must contain the most important information of the data. This information often means consistent features of the input dataset, and finally, this kind of information is gathered into a single manifold. Here, we can use the least squares estimation, assuming that the differences between inputs and outputs follow a normal distribution. Therefore, the objective function that the model aims to minimize is as follows:

(1) $$L_{\left(x,\hat{x}\right)}=\sum_{i}\left|\right|x_{i}-\hat{x_{i}}{\left|\right|}^{2}$$

The mean of the normal distribution is the prediction of the model since we use the least squares estimation, and we can express it in a certain non-linear combination through a network composed of τ. Therefore, the likelihood of the observation (training data) *x* for certain μ and $\sigma^{2}$ is:

(2) $$p\left(x|\tau \right)=p\left(x|\mu ,\sigma \right)=\prod_{i=1}^{n}\frac{1}{\sqrt{2\pi \sigma^{2}}}e^{\frac{-{\left(x_{i}-\mu \right)}^{2}}{2\sigma^{2}}}$$

Finding the network parameters to minimize Formula (1) is, in fact, equivalent to maximizing the likelihood in Formula (2). Note that log is monotone and can be used for the log-likelihood to differentiate them easily. We can detect that the general auto-encoder still performs the maximum likelihood scheme like any other probabilistic model. Although there is no prior distribution, such as an exponential family to control the decoder, the encoder network can abstract the given data to a latent space where the low-dimensional manifold exists, with the dimensionality reduction. The decoder network can also interpret the given abstraction and reconstruct it. This entire process is called “auto-encoding”, which allows at least the networks to be able to learn the consistent features of training data by regularizing them with manifold learning. Since we use the auto-encoder parameterized by τ to output $\hat{x}$, the parameters can be divided into two sets: the encoder network and the decoder network. We denote them as $f_{\phi }$ and $g_{\theta }$, respectively, and the training algorithm is described in Algorithm 1. The training algorithm of the model minimizes the mean squared error and iteratively updates the parameters of the given network. The parameters are initialized by Xavier [22] before training the model, and then, the parameters are updated after computing the mean squared error until it is qualitatively converged using the stochastic gradient descent method.

**Algorithm 1:** Auto-encoder training algorithm.**Input:** Sets of data to train $x_{1},x_{2}\ldots x_{n}$ **Output:** encoder $f_{\phi }$, decoder $g_{\theta }$  ϕ,θ← Initialize network parameters **repeat**  Compute mean squared error  $L_{\left(\phi ,\theta ;x_{i}\right)}=\sum_{i}\left|\right|x_{i}-g_{\theta }\left(f_{\phi }\left(x_{i}\right)\right){\left|\right|}^{2}$  ϕ,θ← Update parameters by SGD **until** convergence of parameters (ϕ,θ)

### 2.3. Abnormality Decision

**Figure 6:**
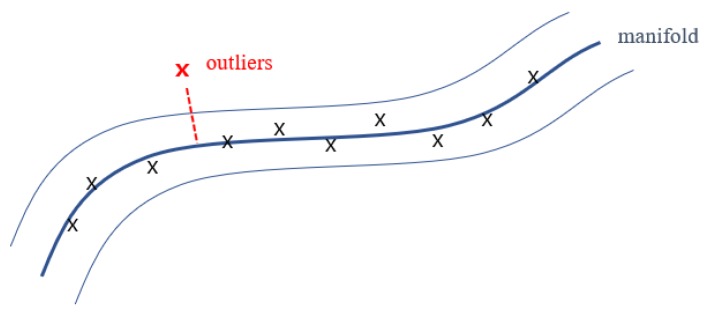
Low-dimensional manifold composed of normal data of the model trained only with them. The outliers cannot be mapped into the manifold properly due to different features from normal data.

The auto-encoder minimizes the loss of information during compression and expansion. Since the high-frequency components are lost in the compression process of the encoder, they are not clearly restored, even if the decoder restores them to the maximum. The objective function of the network is to reduce the average difference between input and output spectrograms, and the element-wise reconstruction is not completely equivalent to its original input. However, in this auto-encoding process, we rather focus on the difference in reconstruction quality when the input is “normal” or “abnormal”. The model learns the important features of the given data, and the low-dimensional vector, which is a result of dimensionality reduction, implies the most significant (highly abstracted) information. Formalizing this approach as learning specific consistent features in normal data, we can use the low-dimensional manifold composed of these feature vectors for abnormality decisions, as depicted in Figure 6.

There are two typical cues in this approach. First, the model expects the data that have a quite similar distribution with the normal training data, and secondly, the abnormalities are characterized by somewhat different features from normal. We integrate these in the auto-encoding steps. The outliers cannot be well mapped to the low-dimensional manifold formed by the normal data, and they can be detected by the property of the decoder that cannot restore them clearly. The distinction between the abnormal data and normal data is defined as the difference between the input and output of a certain level or more. Thus, Formula (1) is an anomaly determinant for unseen data while being the objective function in the training process. This determinant can be an indicator of how close the given data are to the outliers, and whether the data are approaching the outliers. After successfully training the auto-encoder to learn the low-dimensional manifold of normal data, the algorithm detecting the anomalies is described in Algorithm 2.

In residual-error based anomaly detection algorithm, threshold α is determined by using the results for the validation set in the same normal data, but reflecting several abnormal data is more adaptive to selecting an optimal cutoff. Due to the nature of the machine, aging is inevitable and necessary to adjust the threshold for the anomaly determinant as time goes by. The manifold shape of the latent variable *z* of the existing model is slightly deformed. Therefore, it becomes more adaptive when we further train the model with new data. The detection rate varies depending on α; however, it can also be adjusted according to the number of samples used for the anomaly determinant. More details will be described in the next section.

**Algorithm 2:** Residual error-based anomaly detection algorithm.**Input:** Sets of data to classify $x_{1},x_{2}\ldots x_{n}$ **Output:** Residual error $L_{\left(x,\hat{x}\right)}$  ϕ,θ← train network parameters with Algorithm 1  α← set threshold based on training set **repeat**  **for i=1 to N do**    Compute residual error $L_{\left(x,\hat{x}\right)}$    $L_{\left(\phi ,\theta ;x_{i}\right)}=\sum_{i}\left|\right|x_{i}-g_{\theta }\left(f_{\phi }\left(x_{i}\right)\right){\left|\right|}^{2}$    **if** residual error $L_{\left(x,\hat{x}\right)}>\alpha$
**then**      $x_{i}$ is abnormal    **else**      $x_{i}$ is normal    **end if**  **end for**

## 3. Experiment

The data to be classified as abnormal or normal consist of machine operation sounds. In this section, we introduce our very complex experimental data source, a Surface-Mount Device (SMD) assembly machine, which performs a dozen operations in a second, and it will be further described with our results. Note that this residual error-based anomaly detection cannot be experimentally compared with the general classification models. The experimental environment in this paper does not include abnormal data in the training process. Using abnormal data for the abnormality decision has different conditions, which is not the same environment. However, the abnormal data that we have can be exploited in semi-supervision, and this will be discussed in the next section.

### 3.1. SMD

**Figure 7:**
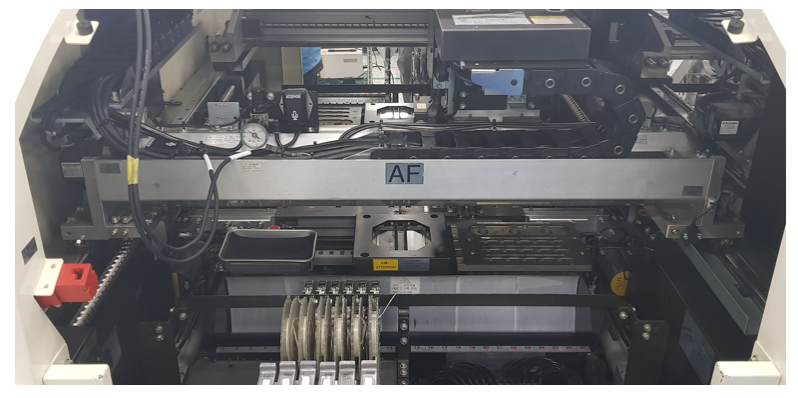
SMD semiconductor assembly equipment.

An SMD is an electronic device (see Figure 7) made using Surface-Mount Technology (SMT). The produced devices are electronic circuits where the components are mounted on the surface of the PCB board. This method provides a fast manufacturing process, but a risk of defects always exists due to denser, miniaturized components. While existing sound-based anomaly detection research is monotonous, the SMD machine is relatively complicated. It can also get more complicated in the sense that the pattern can change according to the component that is assembled. Thus, our experimental data, the SMD machine sound, are considered to be quite complex and more challenging than other machines where the operations are quite monotonous and simple.

The entire production line in our experimental data consists of the C-line and B-line. Both lines are the names set by the company who provided these datasets, and they are different in terms of which semiconductors are being assembled. We selected one production C-line as a normal category and assumed all others to be abnormal (one vs. all). Abnormal refers to everything that is not normal. To test the proposed model, we manually defined three abnormal categories: changing assembly part (from C-Line to B-line), adding noise artificially (to represent broken internal components) and removing grease in the same production C-line. In particular, a non-greased production line requires precise detection because the operating sound is very similar to the normal sound, and it shows a slight difference near the joints of the machine.

**Figure 8:**
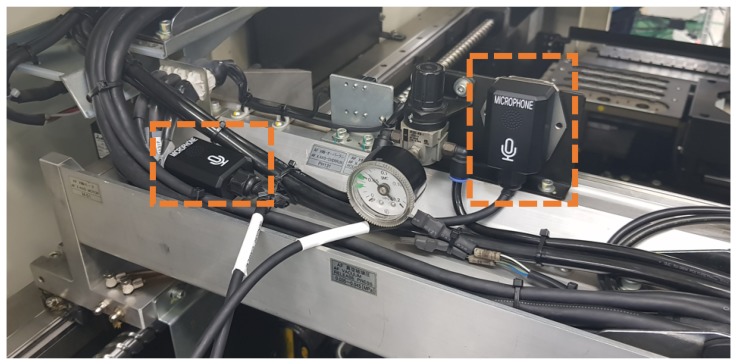
Data collection with microphones. They were attached to each side of the machine joint and the hydraulic cylinder.

The machine operating sound is recorded by attaching the recording sensor to the machine being monitored, as shown in Figure 8. Since the audio data are recorded from a fixed space and closely attached inside the machine, additional processing for the ambient noise is not required. The C-line is the most important normal data in our approach, and data collection is ongoing after the experiment. Therefore, the proposed model becomes more robust once more data are collected. Another category of abnormalities, as previously introduced, consists of the differences in the assembly parts (B-line), non-greased C-line and C-line with intermittent noise. In the case of intermittent noise, we manually added a clacking noise in the recording. We consider the non-greased C-line as an abnormal category because it has no significant problems during operation, but it is likely to be potentially broken down. Since the proposed model only regresses normal data, the abnormal category datasets are used for testing purposes only. The sample dataset is available at https://github.com/DongYuls/SMD_Anomaly_Detection and the total length for each category is shown in Table 1.

For our data preprocessing (see Figure 3), 2048 samples were transformed to a frequency domain with a 512-sample overlap length. Therefore, the column of the input matrix already contained temporal information for 2048 samples. However, anomalies differ not only in the frequency domain, but also in the time domain along their actions; i.e., the joints can be broken or can produce strange behavior over a long period of time.

(3) $$S_{period}=\frac{window\;size+(width-1)\cdot (window\;size-overlap\;length)}{sampling\;rate}$$

Since the size of the spectrogram is only determined by the windowing size in STFT and the audio sampling rate, we can easily determine how long we monitor the machine. Note that the maximum frequency of the STFT cannot exceed half the sampling rate. We can still maximize the frequency resolution by increasing the window size; however, the time resolution would decrease, which makes it difficult to detect the temporal anomaly or intermittent noise. Thus, the column size for each segmentation was experimentally determined as previously introduced in Section 2.1; 32 columns, and represents about 1.5 s considering a sampling rate of 16,300 Hz and 512 samples for the overlap length in our implementation. We obtain 2100 segments in C-line as the total amount of the experiment. The following is the time length represented by the spectrogram according to the hyperparameter of the STFT.

### 3.2. Result

**Figure 9:**
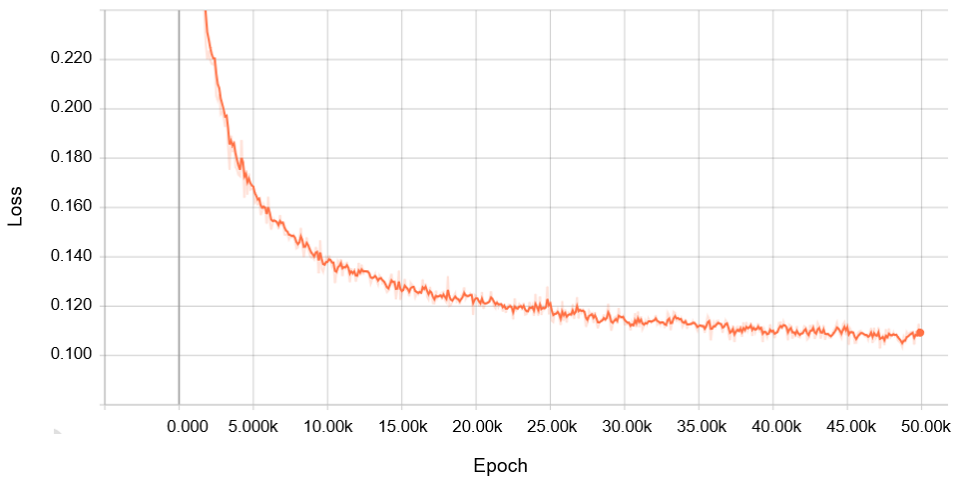
Results of the training process (Adam optimizer with learning rate = 0.0008, batch size = 32) for a normal assembly C-line, using GTX 1060 for 50 K steps and taking 8 h.

We regarded the C-line as a normal class, and the others including grease-removed C-line, B-line and artificial-noise addition in both lines as an abnormal class. We also split the C-line dataset into 2000 segments for the training set and 100 segments for the validation set. Note that we do not use abnormal data in the training process, but instead use them to test whether the model outputs high residual errors. The results of training our model are depicted in Figure 9.

**Figure 10:**
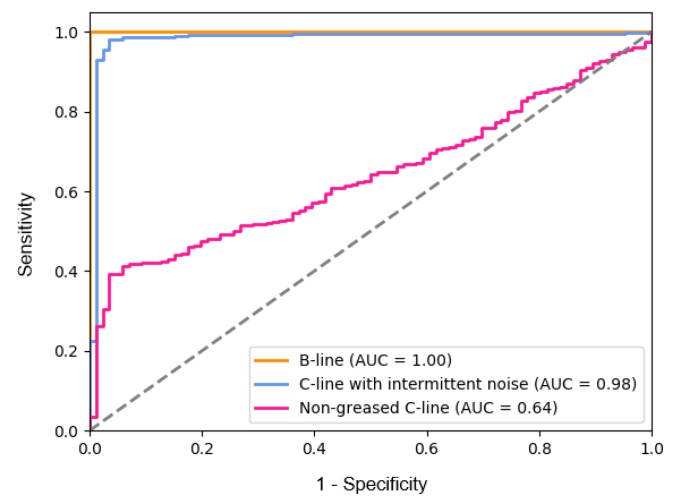
Receiver Operation Characteristic (ROC) curve of the proposed method for each category corresponding to Table 1. The accuracy is measured by the Area Under the ROC Curve (AUC). Note that this is not a multi-classification result, but an individual ROC curve with a normal C-line.

After training the model, we measured the distribution of the reconstruction errors for normal and abnormal data before defining the threshold (refer to Algorithm 2). We had experiments to assess the proposed approach on a single category, which is whether the model really distinguishes abnormal and normal sounds. According to our anomaly detection method, the threshold α was adaptively set based on the histogram of the reconstruction error for the validation set. However, we do not use the maximum value in the histogram as a threshold due to the trade-off between the sensitivity and the specificity, as depicted in Figure 10. The supervisor’s demand is fully reflected in terms of the optimal cutoff. That is, we can maximize the abnormal detection rate (high sensitivity) to ensure that the machine constantly remains in a normal state, even if there are frequent false alarms due to a low specificity.

We observed (refer to Figure 10) that the performance of the distinction between the non-greased C-line and normal C-line is poor, while the intermittent sound is easily distinguishable because it is rarely observed in normal operating sound. The B-line is also very easy to distinguish as the assembly part changes since all operations, such as mounting and air rejection, are different. However, the non-greased C-line can be thought of not only as a situation that is easily observed from an environment related to the machine, but also as an indication of aging.

**Figure 11:**
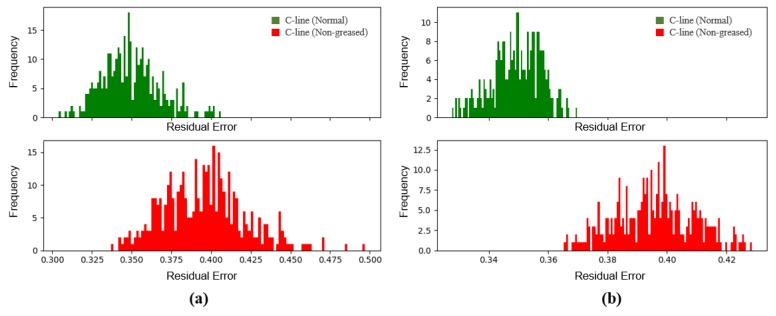
Histogram of the average residual errors between the input data and its reconstruction by the auto-encoder: (**a**) the average error of single samples; (**b**) the average error of 20 samples. Note that abnormal data shown above represent the non-greased C-line.

In order to improve the performance on a non-greased C-line, we considered two observations, which cause the fault detection using a single segment to produce many false alarms, as depicted in Figure 11. First, the sound is identical regardless of whether it is greased or not in certain operations. For example, since cylinders in the machine do not contain the grease, their sounds are independent of the grease. We did not remove or process them in the training set, because there is no prior knowledge of the abnormalities, and the machine does not always show an indication of them in all sections. We applied the model to a more general situation where the abnormal noise and the normal operation sound both exist. Second, the non-greased C-line and normal C-line have exactly the same operations, but different sounds during the operations due to different frequency bands by the presence of grease. We observed that grease makes a slight difference in low frequency bands, and it is quite challenging for the model to distinguish them because of the frequency resolution trade-off. Thus, we performed anomaly detection through an ensemble of several samples (segments). Note that the x-axis in Figure 11 shows the residual errors for the average difference between the input spectrogram and the reconstructed spectrogram, according to the number of samples used for the anomaly determinant, and the y-axis shows their frequency.

**Figure 12:**
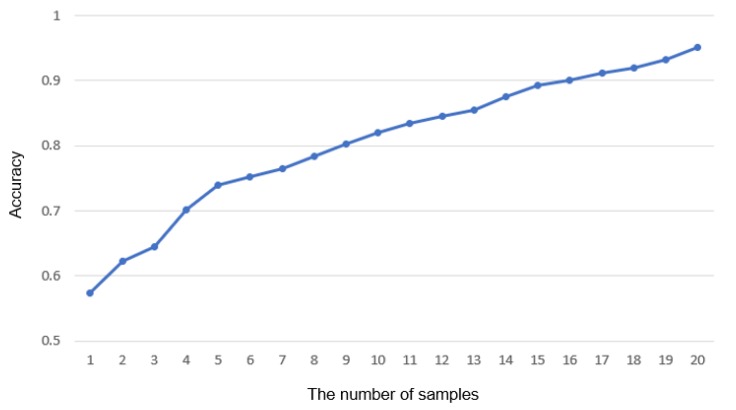
The variation in accuracy according to the number of samples used as a determinant for a normal C-line. The entire audio data recorded in the abnormal line contain the features independent of being abnormal and normal, such as the rest interval.

The noise caused by colliding internal components or severe damage of a gear can be detected in real time, whereas the noise caused by aging cannot. This is because aging is a natural phenomenon due to the nature of the machine, and it only shows a slight difference in frequency from normal operation sound. However, the SMD managers also claim that this type of abnormality does not need real-time service, since it does not cause serious defects in the production line immediately and can be solved by the maintenance of the manager. As the number of samples in Figure 12 increases, the accuracy will reach 100%. Consequently, it takes a longer time to detect the anomalies, and the more short-lived anomalies can be neglected. We conclude that the number of the optimal samples in actual use is 20. All these can be adjusted by the manager’s opinion for maintenance.

## 4. Conclusions

Detecting whether a machine is approaching an abnormal state or not is more meaningful than just distinguishing what is already broken. Therefore, it is possible to detect slight changes in the frequency before a more severe fault occurs and to prepare measures against aging. The proposed approach showed a high performance in detecting anomalies, and it can be applied to more general situations and help solve the data imbalance problem. That is, we have created an auto-encoding manifolder that can measure the differences without expensive annotations for anomalies.

In addition, we successfully detected anomalies using a cost-effective modality. The approach of conventional models is mostly based on repeated-deterministic signals. However, our approach can handle stochastic signals. In other words, the general classification model outperforms others if there is prior knowledge of the situation, such as a limited variety within sound events. The key method of the proposed model is the neural network-based regression analysis within the auto-encoder framework. It can be used not only in the SMD machine, but also in various situations or in environments where data acquisition is difficult, for example, in the medical field, people who are sick or cannot be considered normal and abnormal, and their abnormal labels are difficult to obtain because only experts or doctors can make a diagnosis through data.

Once having detecting anomalies or outliers, we need to classify the anomalies. The ensemble with the operation-classification model based on semi-supervised learning techniques can be suitable to further improve the performance by tracking the area where anomalies occur. In addition, tactile and visual aspects of an object are the main modalities to define the object, as well as the auditory aspect. This research was initiated by the SMD manufacturer’s opinion that the manager can distinguish the abnormalities by the operation sound. It is possible to extend it to a visual or vibration sensor in a similar way. In future work, we would like to explore the semi-supervision framework and the ensemble with other modalities. 

## Figures and Tables

**Table 1:** Dataset of audio records in the experiment.

Category	Length
C-line	24:49
Non-greased C-line	15:53
C-line with intermittent noise	06:08
B-line	11:00

## Supplementary Materials

The following are available online at http://www.mdpi.com/1424-8220/18/5/1308/s1.

## Appendix A

**Table A1:** Implementation Detail.

Operation	Kernel	Stride	Feature Maps	Batch-Norm.	Activation-Fn.
Encoder Input: 1024 × 32 × 1					
Convolution	5 × 5	2 × 1	64	Yes	ReLU
Convolution	5 × 5	2 × 1	64	Yes	ReLU
Convolution	5 × 5	2 × 1	96	Yes	ReLU
Convolution	5 × 5	2 × 1	96	Yes	ReLU
Convolution	5 × 5	2 × 1	128	Yes	ReLU
Convolution	4 × 4	2 × 2	128	Yes	ReLU
Convolution	4 × 4	2 × 2	160	Yes	ReLU
Convolution	4 × 4	2 × 2	160	Yes	ReLU
Convolution	3 × 3	2 × 2	192	Yes	ReLU
Convolution	3 × 3	2 × 2	192	No	None
Decoder Input: 1 × 1 × 192					
Convolution	3 × 3	2 × 2	192	Yes	ReLU
Convolution	3 × 3	2 × 2	160	Yes	ReLU
Convolution	4 × 4	2 × 2	160	Yes	ReLU
Convolution	4 × 4	2 × 2	128	Yes	ReLU
Convolution	4 × 4	2 × 2	128	Yes	ReLU
Convolution	5 × 5	2 × 1	96	Yes	ReLU
Convolution	5 × 5	2 × 1	96	Yes	ReLU
Convolution	5 × 5	2 × 1	64	Yes	ReLU
Convolution	5 × 5	2 × 1	64	Yes	ReLU
Convolution	5 × 5	2 × 1	1	No	None

Activation function represents a type of non-linear combination as passing each layer. Batch-normalization prevents internal covariance shift, which refers to the variation of input distribution with each non-linearity in the network. More details about batch-normalization can be found in [23].
